# Oliguria as predictive biomarker of acute kidney injury in critically ill patients

**DOI:** 10.1186/cc10318

**Published:** 2011-07-19

**Authors:** John R Prowle, Yan-Lun Liu, Elisa Licari, Sean M Bagshaw, Moritoki Egi, Michael Haase, Anja Haase-Fielitz, John A Kellum, Dinna Cruz, Claudio Ronco, Kenji Tsutsui, Shigehiko Uchino, Rinaldo Bellomo

**Affiliations:** 1 Department of Intensive Care, Austin Hospital, 145 Studley Road, Heidleberg, Victoria 3084, Australia; 2 Division of Critical Care Medicine, University of Alberta, 3C1.12 Walter C. Mackenzie Centre, 8440-122 Street, Edmonton, AB T6G2B7, Canada; 3 Department of Anesthesiology and Resuscitology, Okayama University Medical School, 5-1 Shikata-Cho 2-Chome, Okayama 700-8558, Okayama, Japan; 4 Department of Nephrology and Intensive Care Medicine, Charité University Medicine, 1 Augustenburger Platz, Berlin 13353 Germany; 5 The Clinical Research, Investigation, and Systems Modeling of Acute illness (CRISMA) Center, Department of Critical Care Medicine, University of Pittsburgh, School of Medicine, 3550 Terrace Street, Pittsburgh, PA 15261, USA; 6 Department Nephrology Dialysis & Transplantation San Bortolo Hospital. International Renal Research Institute (IRRIV), Vicenza, Italy; 7 Intensive Care Unit, Department of Anesthesiology, Jikei University School of Medicine, 3-25-8, Nishi-Shimbashi, Minato-ku, Tokyo, 105-8461, Japan; 8 Australian and New Zealand Intensive Care Research Centre, School of Public Health and Preventive Medicine, Monash University, 3004 Melbourne, Victoria, Australia

**Keywords:** Oliguria, Kidney Failure, Acute, Critical Illness, creatinine, urine, biomarkers

## Abstract

**Introduction:**

During critical illness, oliguria is often used as a biomarker of acute kidney injury (AKI). However, its relationship with the subsequent development of AKI has not been prospectively evaluated.

**Methods:**

We documented urine output and daily serum creatinine concentration in patients admitted for more than 24 hours in seven intensive care units (ICUs) from six countries over a period of two to four weeks. Oliguria was defined by a urine output < 0.5 ml/kg/hr. Data were collected until the occurrence of creatinine-defined AKI (AKI-Cr), designated by RIFLE-Injury class or greater using creatinine criteria (RIFLE-I[Cr]), or until ICU discharge. Episodes of oliguria were classified by longest duration of consecutive oliguria during each day were correlated with new AKI-Cr the next day, examining cut-offs for oliguria of greater than 1,2,3,4,5,6, or 12 hr duration,

**Results:**

We studied 239 patients during 723 days. Overall, 32 patients had AKI on ICU admission, while in 23, AKI-Cr developed in ICU. Oliguria of greater than one hour was significantly associated with AKI-Cr the next day. On receiver-operator characteristic area under the curve (ROCAUC) analysis, oliguria showed fair predictive ability for AKI-Cr (ROCAUC = 0.75; CI:0.64-0.85). The presence of 4 hrs or more oliguria provided the best discrimination (sensitivity 52% (0.31-0.73%), specificity 86% (0.84-0.89%), positive likelihood ratio 3.8 (2.2-5.6), *P *< 0.0001) with negative predictive value of 98% (0.97-0.99). Oliguria preceding AKI-Cr was more likely to be associated with lower blood pressure, higher heart rate and use of vasopressors or inotropes and was more likely to prompt clinical intervention. However, only 30 of 487 individual episodes of oliguria preceded the new occurrence of AKI-Cr the next day.

**Conclusions:**

Oliguria was significantly associated with the occurrence of new AKI-Cr, however oliguria occurred frequently compared to the small number of patients (~10%) developing AKI-Cr in the ICU, so that most episodes of oliguria were not followed by renal injury. Consequently, the occurrence of short periods (1-6 hr) of oliguria lacked utility in discriminating patients with incipient AKI-Cr (positive likelihood ratios of 2-4, with > 10 considered indicative of a useful screening test). However, oliguria accompanied by hemodynamic compromise or increasing vasopressor dose may represent a clinically useful trigger for other early biomarkers of renal injury.

## Introduction

Urine output monitoring is almost universal in critically ill patients worldwide. Historically, maintenance of urine output has been regarded as being synonymous with the preservation of renal function and decreases in urine output regularly prompt a variety of clinical interventions with the aim of preventing or attenuating acute kidney injury (AKI) [1]. According to this paradigm, after exclusion of obstruction, decreased urine output is considered a clinically useful biomarker of decreased glomerular filtration rate (GFR), which occurs before the detectable accumulation of biochemical markers of AKI. As a result, consensus definitions of AKI have incorporated urine output criteria alongside biochemical markers of renal excretory function [2,3]. Unfortunately, this step has occurred despite a lack of any prospective data in critically ill patients associating the magnitude and duration of oliguria with subsequent changes in serum biochemistry. In addition, urine output criteria used in consensus definitions have ignored the potential value of shorter periods of oliguria.

It is important to note that Risk, Injury, Failure, Loss, End-Stage (RIFLE) and Acute Kidney Injury Network (AKIN) consensus definitions of AKI incorporate lengthy periods of oliguria (6, 12, and 24 hours) by which time significant renal injury may have already occurred. Consequently, much shorter durations of oliguria, such as two hours or less [4], have been recommended and are often used as targets for therapeutic intervention. It is uncertain, however, how many patients experiencing shorter periods of oliguria are at significant risk of biochemical AKI defined by creatinine criteria (AKI-Cr). Furthermore, it is unclear how sensitive any duration of oliguria is at identifying AKI-Cr given that urine output can be relatively preserved even when GFR is significantly impaired [5,6]. Accordingly, we hypothesized that oliguria would only be a poor to fair predictive biomarker of subsequent AKI-Cr. To test this hypothesis, we performed a prospective multicenter observational study of the relation between oliguria and subsequent AKI-Cr in a cohort of critically ill patients in seven centers worldwide.

## Materials and methods

### Study population and methodology

Local Ethics Committee approval was obtained for anonymous analysis of routinely collected clinical data with waiver of informed consent. A case report form was generated centrally and distributed to experienced center lead investigators who supervised the local data-collection process. We documented hourly urine output and daily serum creatinine (sCr) in unselected consecutive ICU admissions of at least one calendar day in seven adult ICUs from six countries over periods of two weeks (six centers) or four weeks (one center) during 2008. Patients with end stage renal disease (on maintenance dialysis) or without a urinary catheter were excluded. Patient demographics, admission clinical details, Simplified Acute Physiology Score (SAPS II) illness severity scoring [7] on ICU admission and biochemistry were also documented. Patients were defined as medical or surgical by nature of admitting clinical service. Sepsis during ICU admission was defined as the presence of a systemic inflammatory response syndrome (SIRS) response in the context of proven or suspected infection [8]. Following RIFLE and AKIN criteria, oliguria was identified as consecutive hours of urine output of less than 0.5 ml/kg body weight. AKI-Cr was defined as new RIFLE Injury or greater, using the sCr criteria of the RIFLE consensus definition - a doubling of sCr from pre-morbid baseline (RIFLE I[Cr]). The intention of this study was to assess the ability of varying periods of reduced urine output to predict biochemical evidence of renal dysfunction. Thus, only creatinine criteria of the RIFLE consensus definition of AKI were used to define outcome. Where pre-morbid biochemical data was not available, a baseline sCr was estimated assuming a GFR of 75 ml/min as previously described [9]. One patient received renal replacement therapy (RRT) for ongoing anuria prior to meeting biochemical criteria for RIFLE-I and was analyzed as developing AKI-Cr. Data were collected until the occurrence of AKI-Cr defined as RIFLE I[Cr] or greater or ICU discharge, oliguria during days in ICU after the occurrence of AKI-Cr were not included. Episodes of oliguria during each 24-hour period were correlated with the occurrence of new RIFLE I[Cr] or greater on routine morning bloods the next day. We considered that more sustained oliguria may reflect extended periods of renal hemodynamic compromise and might better predict biochemically evident renal dysfunction. Thus, we sought to correlate the maximum duration of consecutive oliguria on any given ICU day with the occurrence of new RIFLE I[Cr] or greater on routine bloods the next morning. We secondarily assessed the predictive ability of oliguria in each of the two days preceding AKI-Cr and repeated our original analysis limiting data to that collected on the first three ICU days only.

For each individual episode of oliguria basic hemodynamic variables at the beginning of the episode and any clinician response to oliguria (fluid, vasoactive drug, or diuretic prescription) were recorded and comparison was made between episodes of oliguria that were and were not associated with progression to RIFLE I[Cr] the next day. In this analysis individual episodes of oliguria were treated as discrete events. This is because two different episodes of oliguria occurring on the same day might have occurred in different hemodynamic contexts and prompted different interventions. Clinical interventions were deemed to be associated with oliguria if they occurred during or within one hour of the end of a period of oliguria.

### Data handing and statistical analysis

Data were collected and collated using Microsoft Excel (Microsoft Corp, Redmond, WA, USA). Categorical variables were compared using Fisher's exact test, continuous data were reported as median with inter-quartile range (IQR) and compared using the Mann-Whitney U test. Receiver-operator characteristic (ROC) curve analysis was used to assess the ability of varying duration of the longest period of oliguria to predict the occurrence of RIFLE I[Cr] or greater the next day. Univariate statistics, ROC curve analysis and area under curve (AUC) calculation was carried out using GraphPad Prism version 5.0 d for Mac OS (GraphPad Software, La Jolla, CA) [10] additionally binomially fitted ROC curves and asymmetric 95% confidence intervals were prepared using ROC analysis: web-based calculator for ROC curves [11]. We defined an ROCAUC of 0.5 to 0.6 as showing no predictive ability, an ROCAUC of 0.6 to 0.7 as showing poor predictive ability, an ROCAUC of 0.7 to 0.8 as showing fair predictive ability, an ROCAUC of 0.8 to 0.9 as showing good predictive ability, and an ROCAUC above 0.9 as showing outstanding predictive ability for AKI-Cr.

## Results

### Patient characteristics

We studied 239 patients during 723 ICU days (Table 1). Pre-morbid sCr was available in 59.8% of patients and was estimated in 40.2%. Weight was measured at hospital admission in 69.5% and estimated in the ICU by local protocol in 30.5%.

**Table 1:** Patient characteristics

	**No**.	Male	Surgical	Sepsis	SAPS 2	AKI on admission	AKI in ICU	RRT	28-Day mortality
**Australia**	88	58%	57%	34%	31	11	10	6	15%
**Canada**	31	59%	25%	68%	45	13	0	7	22%
**Japan 1**	16	69%	75%	19%	31	1	2	1	0%
**Japan 2**	15	40%	93%	6%	25	0	0	0	6.3%
**USA**	34	50%	12%	32%	*na*	3	1	3	12%
**Germany**	35	66%	17%	37%	34	4	5	4	8.5%
**Italy**	19	74%	100%	0%	29	0	5	0	0%
**All**	239	59%	48%	34%	31	32	22	21	11%

Study centers: Austin Hospital, Heidelberg, Victoria, Australia (R. Bellomo), University of Alberta Hospital, Calgary, Alberta, Canada (S. Bagshaw), Charité University Medicine, Potsdam, Germany (M. Haase), Okayama University Hospital, Shikata City, Japan (M. Egi), Jikei University School of Medicine, Tokyo, Japan (S. Uchino), UPMC McKeesport, Pittsburgh, PA, USA (J. Kellum) and Ospedale San Bortolo, Vicenza, Italy (C. Ronco).

AKI, acute kidney injury; na, not available; RRT, renal replacement therapy; SAPS 2, simplified acute physiology score 2.

Overall, 28-day mortality was 10.7% and 54 (22.5%) patients had sCr criteria for RIFLE I[Cr] or greater. Of these, 32 (13.4%) had AKI-Cr on ICU admission and were not considered further, whereas in 23 individuals (9.6%), new AKI-Cr developed in ICU. Overall, of the 293 patients, 21 (8.8%) received RRT in the ICU. Of the 54 patients with AKI-Cr, 21 (39%) received RRT; however, of the 23 patients developing AKI-Cr in the ICU only five received RRT, this is 22% of AKI-Cr in ICU. On the other hand, 50% of patients with AKI-Cr on admission required RRT. The median day of AKI-Cr occurring in the ICU was day three (range second to fourth ICU day). The relation between type of admission (medical/surgical), or diagnosis of sepsis and the occurrence of AKI-Cr on admission or in the ICU is shown in Table 2. Significantly more patients with AKI-Cr on ICU admission had a diagnosis of sepsis (*P *= 0.015) while more patients who developed AKI-Cr in the ICU were surgical than medical (*P *= 0.05).

**Table 2:** Relation between admitting service and sepsis diagnosis with AKI-Cr on ICU admission and AKI-Cr in the ICU

	All Admissions	Admissions ith AKI-Cr	*P*	Admissions without AKI-Cr	AKI-Cr in ICU	*P*
**Medical**	125	19 (15%, 9-22%)	0.45	106	7 (5.6%, 1.5-10%)	0.05
**Surgical**	114	13 (11%, 5-17%)		101	16 (14%, 8-21%)	
**Sepsis**	80	17 (21%, 12-30%)	0.015	63	9 (11%, 4-18%)	0.35
**No Sepsis**	159	15 (9.4%, 5-14%)		143	14 (8.8%, 4-13%)	

Percentages are percent patients developing AKI of all admissions in that category with 95% confidence intervals. AKI-Cr, acute kidney injury defined by changes in serum creatinine.

### Oliguria occurring prior to diagnosis of AKI-Cr

Episodes of oliguria of one hour or more occurred on 265 of 723 study days and were significantly associated with the occurrence of new AKI-Cr on the next day (Table 3). However, on 257 days (38%), oliguria of one hour or more occurred without progression to RIFLE of one or more the next day. Most episodes of oliguria, regardless of duration, were not closely followed by renal injury. Indeed, on 9 of 13 occasions, greater than 12 hours of consecutive oliguria (oliguria ≥12 hour) (RIFLE I by urine output criteria) occurred without development of RIFLE-I by sCr criteria the next day (Table 3). Many patients developing AKI-Cr did not have prolonged periods of oliguria on the day prior to diagnosis of AKI-Cr, with only 52% (12 of 23) of such patients experiencing oliguria for four or more hours during the preceding day and 5 of 22 patients progressing to RIFLE I without any oliguria the preceding day.

**Table 3:** Relation between length of longest episode of oliguria during an ICU day at risk (patient day without a diagnosis of RIFLE I[Cr]) and AKI-Cr the next day

Longest duration of oliguria	Days with AKI-Cr next day	Days with no AKI-Cr next day	**Sens**.	**Spec**.	PPV	NPV	LR	*P*	RR of AKI-Cr
**None**	5	443							
**≥1 hr**	18	257	0.78	0.63	0.07	0.99	2.1	< 0.0001	5.9
**≥2 hr**	15	194	0.65	0.72	0.07	0.98	2.4	0.0003	4.6
**≥3 hr**	13	125	0.57	0.82	0.09	0.98	3.2	< 0.0001	5.5
**≥4 hr**	12	95	0.52	0.86	0.11	0.98	3.8	< 0.0001	6.3
**≥5 hr**	7	75	0.30	0.89	0.09	0.98	2.8	0.01	3.4
**≥6 hr**	5	50	0.21	0.93	0.09	0.97	3.1	0.02	3.8
**≥12 hr**	4	9	0.17	0.99	0.31	0.97	13.5	0.0005	11.5

*P*-values refer to Fisher's exact test for presence or absence of stated degree of oliguria and AKI outcome.

AKI-Cr, acute kidney injury defined by changes in serum creatinine; hr, hours; LR, likelihood ratio (positive); NPV, negative predictive value; PPV, positive predictive value; Sens, sensitivity; Spec, specificity; RR, relative risk.

### Ability of oliguria to predict AKI-Cr

**Figure 1:**
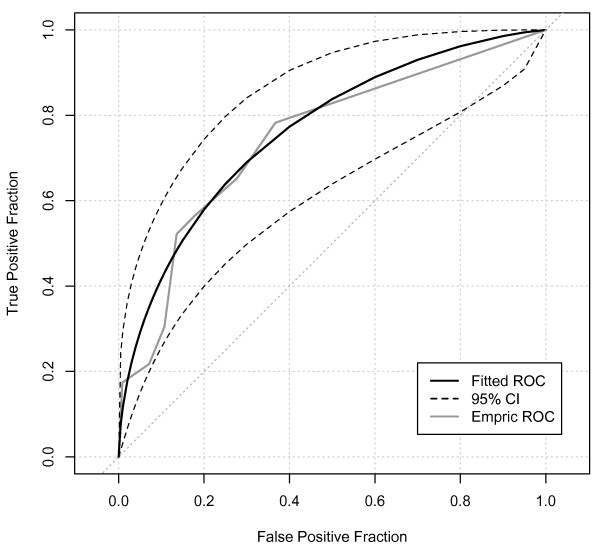
**Receiver-operator characteristic analysis of the ability of varying durations of oliguria to predict RIFLE Injury (I) or more the next day**. Receiver-operator characteristic (ROC) area under the curve = 0.75, 95% confidence interval (CI) 0.64-0.85.

**Figure 2:**
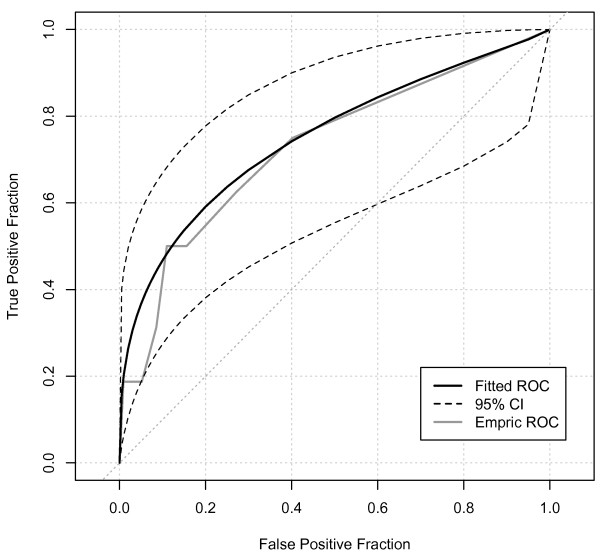
**Receiver-operator characteristic analysis of surgical patients**. Receiver-operator characteristic (ROC) area under the curve = 0.73, 95% confidence interval (CI) 0.59-0.87.

**Figure 3:**
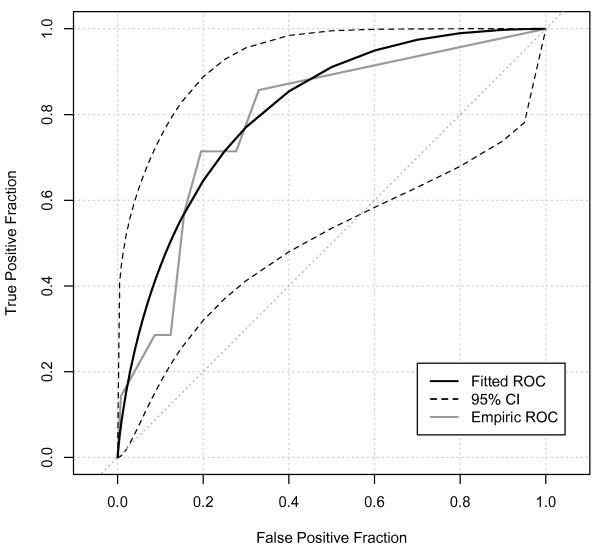
**Receiver-operator characteristic analysis of medical patients**. Receiver-operator characteristic (ROC) area under the curve = 0.79, 95% confidence interval (CI) 0.63-0.94).

**Figure 4:**
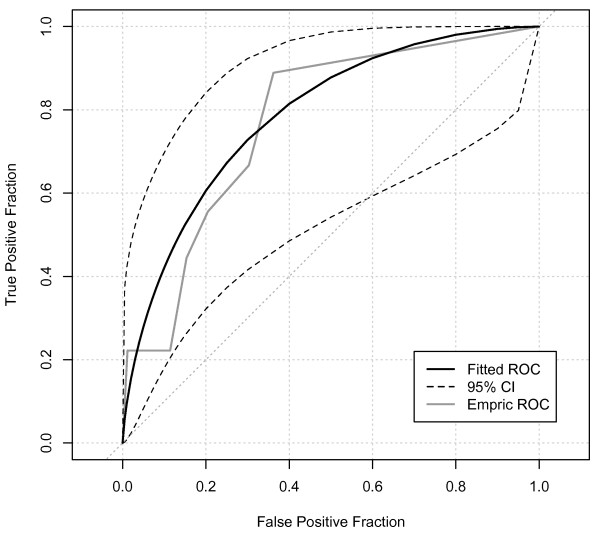
**Receiver-operator characteristic analysis of patients with a diagnosis of sepsis**. Receiver-operator characteristic (ROC) area under the curve = 0.78, 95% confidence interval (CI) 0.64-0.90).

The ROCAUC for oliguria as a predictor of subsequent AKI-Cr (Figure 1) showed a statistically significant, but only fair performance (ROCAUC = 0.75, 95% confidence interval (CI) 0.64-0.85). On balance, oliguria of four or more hours provided best overall combination of sensitivity and specificity (Sensitivity 52%, 95% CI 0.31-0.73; Specificity 86%, 95% CI 0.84-0.8; positive likelihood ratio 3.8, 95% CI 2.2-5.6; *P *< 0.0001). However, even with this degree of oliguria, the positive predictive value for AKI-Cr was only 11% (95% CI 6-19%; Table 3). For all levels of oliguria, there were strong negative predictive values (all > 97%) reflecting the low daily incidence of AKI-Cr. When analyzing medical and surgical ICU admissions separately, AKI-Cr in the ICU was more common in surgical patients (Table 2). However, oliguria appeared no better at predicting AKI-Cr in surgical admissions (ROCAUC = 0.73, 95% CI 0.59-0.87, Figure 2) than in medical patients (ROCAUC = 0.79, 95% CI 0.63-0.94, Figure 3). Similarly, there was no significant difference in predictive ability for admissions with sepsis (ROCAUC = 0.78, 95% CI 0.64-0.90, Figure 4). Estimated baseline creatinine was used to define AKI-CR in 40% of patients, this may have led to an over- or under-estimate of the incidence of AKI-Cr in these patients. However, exclusion of all individuals with estimated baseline creatinine did not improve the diagnostic ability of oliguria to predict AKI-Cr in ROC analysis with a ROCAUC of 0.71 (0.54-0.87) when considering only patients with documented baseline creatinine.

Extension of the time period during which the occurrence of oliguria could predict AKI-Cr from the day preceding to the two days preceding AKI-Cr did not improve the ability of oliguria to discriminate AKI-Cr (Table 4) and the ROCAUC for this comparison was 0.72 (0.63-0.81).

**Table 4:** Relation between length of longest episode of oliguria during an ICU day at risk (patient day without a diagnosis of RIFLE I[Cr]) and AKI-Cr in the next two days

Longest duration of oliguria	Days with AKI-Cr next 2 days	Days with no AKI-Cr next 2 days	**Sens**.	**Spec**.	PPV	NPV	LR	*P*	RR of AKI
**None**	9	439							
**≥1 hr**	26	249	0.74	0.63	0.09	0.98	2.1	< 0.001	4.7
**≥2 hr**	22	187	0.62	0.72	0.10	0.97	2.3	< 0.001	4.2
**≥3 hr**	18	120	0.51	0.83	0.13	0.97	2.9	< 0.001	4.5
**≥4 hr**	17	90	0.49	0.87	0.16	0.97	3.7	< 0.001	5.4
**≥5 hr**	10	72	0.29	0.90	0.12	0.96	2.7	0.004	3.1
**≥6 hr**	6	49	0.17	0.93	0.11	0.96	2.4	0.04	2.5
**≥12 hr**	4	9	0.11	0.99	0.31	0.96	8.7	0.002	7.0

Receiver-operator characteristic area under the curve for this comparison = 0.72 (0.63-0.81). *P*-values refer to Fisher's exact test for presence or absence of stated degree of oliguria and AKI outcome.

AKI-Cr, acute kidney injury defined by changes in serum creatinine; LR, likelihood ratio (positive); NPV, negative predictive value; PPV, positive predictive value; Sens, sensitivity; Spec, specificity; RR, relative risk.

Most AKI-Cr in the ICU occurred in the first few days of critical illness. Inclusion of repeated measures of urine output over many days of lengthy ICU admission might lead to some stable patients at low risk of AKI being over-represented in the dataset. We repeated our initial analysis including urine output during the first three days of ICU stay only in all patients with a longer length of stay (Table 5). Again, predictive ability of oliguria did not improve in this analysis with an ROCAUC of 0.72 (0.61-0.83) because, even in patients who did not develop AKI-Cr, most days of oliguria occurred early in ICU admission (Tables 3 and 5).

**Table 5:** Relation between length of longest episode of oliguria during an ICU day at risk (patient day without a diagnosis of RIFLE I[Cr]) and AKI-Cr in the next day considering only data gathered during the first three days of ICU admission

Longest duration of oliguria	Days with AKI-Cr next day	Days with no AKI-Cr next day	**Sens**.	**Spec**.	PPV	NPV	LR	*P*	RR of AKI-Cr
**None**	5	238							
**≥1 hr**	18	175	0.78	0.57	0.09	0.98	1.9	< 0.001	4.5
**≥2 hr**	15	130	0.68	0.69	0.10	0.98	2.2	< 0.001	4.3
**≥3 hr**	13	91	0.56	0.78	0.13	0.97	2.6	< 0.001	4.2
**≥4 hr**	12	71	0.52	0.83	0.14	0.97	3.0	< 0.001	4.6
**≥5 hr**	7	59	0.30	0.85	0.11	0.96	2.1	0.06	2.5
**≥6 hr**	5	40	0.22	0.90	0.11	0.95	2.2	0.08	2.4
**≥12 hr**	4	8	0.17	0.98	0.33	0.96	9.0	0.002	7.4

Receiver-operator characteristic area under the curve for this comparison = 0.72 (0.61-0.83). *P*-values refer to Fisher's exact test for presence or absence of stated degree of oliguria and AKI outcome.

AKI-Cr, acute kidney injury defined by changes in serum creatinine; LR, likelihood ratio (positive); NPV, negative predictive value; PPV, positive predictive value; Sens, sensitivity; Spec, specificity; RR, relative risk.

Looking at oliguria occurring at any stage prior to a diagnosis of AKI-Cr in the ICU, in all 207 patients at risk, 130 (63%) experienced at least one episode of oliguria. Only 20 (15%) of these patients actually developed RIFLE I AKI-Cr. Conversely of the 77 patients with no oliguria, only three (3.9%) developed AKI-Cr (sensitivity 87%, 95% CI 0.66-0.97; specificity 40%, 95% CI 0.33-0.47; positive likelihood ratio 1.5, 95% CI 1.1-1.6; Fisher's exact test: *P *= 0.011).

### Individual episodes of oliguria and relation to occurrence of AKI-Cr

A total of 487 individual episodes of oliguria were recorded in patients at risk of AKI-Cr. Only 30 of these were associated with progression to RIFLE I[Cr] AKI-Cr the next day. Duration of oliguria was not significantly greater in those developing AKI-Cr. However, in univariate analysis, patients developing AKI-Cr had higher heart rate, lower blood pressure, higher central venous pressure and were more likely to be on vasopressor or inotropic medication. Oliguria preceding AKI-Cr was also more likely to receive physician intervention with diuretics or fluid therapy (Table 6).

**Table 6:** Comparison of all individual episodes of oliguria between those that were associated with progression to RIFLE I[Cr] and those that were not

	AKI-Cr	No AKI-Cr	*P*
**Episodes of oliguria**	30	457	
**Duration of oliguria**	3 (1-4)	2 (1-3.75)	0.14
**HR**	90 (79-100)	82 (70-95)	**0.02**
**MAP**	75 (65-80)	78 (70-87.5)	**0.01**
**CVP**	11 (10-13)	9 (7-12)	**0.02**
**On vasopressor or inotrope**	65% (47-82)	32% (28-37)	**0.0002**
**Action**			
**None**	17% (3-32)	52% (47-57)	**0.0002**
**Fluid**	43% (25-62)	26% (22-30)	**0.05**
**Diuretic**	47% (28-66)	26% (22-30)	**0.02**
**Increase/start vasopressor or inotrope**	17% (3-31)	24% (17-30)	0.51

Continuous variables reported as medians and inter-quartile range and compared with Mann-Whitney U test. Categorical variables are reported as percentages with 95% confidence interval and compared with Fisher's exact test. AKI-Cr, acute kidney injury defined by changes in serum creatinine; CVP, central venous pressure; HR, heart rate; MAP, mean arterial pressure.

## Discussion

### Statement of key findings

We performed a prospective, multicenter, multinational observational study to assess the association between oliguria and the subsequent development of AKI-Cr in cohort of medical and surgical critically ill adults. We sought to test the hypothesis that oliguria would only be a poor to fair predictive biomarker of subsequent AKI-Cr. We found that more patients presented to the ICU with AKI-Cr rather than develop it in the ICU. We also found that even the shortest duration of oliguria of one hour was significantly associated with the development of AKI-Cr in the ICU. However, AKI-Cr was infrequent and oliguria was relatively common, with some degree of oliguria occurring on one third of days spent in the ICU in patients without a diagnosis of AKI-Cr. This high false-positive rate precluded the use of oliguria alone for the early identification of AKI-Cr. Conversely, many patients developing AKI-Cr did not have prolonged periods of oliguria the day before diagnosis of AKI-Cr, with only half of AKI-Cr patients experiencing oliguria of four hours or more the preceding day and approximately one quarter of 23 patients progressing to AKI-Cr without any oliguria the preceding day. Oliguria preceding AKI-Cr was more likely to be associated with lower blood pressure, higher heart rate, and use of vasopressors or inotropes, and was more likely to prompt intervention suggesting that hemodynamic status and other factors affecting physician decisionmaking might add to the predictive ability of oliguria. However, only 6.2% of episodes of oliguria of one hour or more were associated with AKI-Cr the next day.

### Comparison with previous studies

To our knowledge this is the first study to prospectively assess the value of varying durations of urine output as a predictive biomarker of AKI-Cr in ICU. Thus, its findings cannot be directly compared with previous studies. However, the issue of oliguria in humans has been explored in the past. In particular, modern concepts of oliguria date back to studies from the 1930s and 1940s examining urine output during fasting in normal individuals [12-14]. In these studies, maximal water conservation achieved a minimal urine output of about 500 ml per day in adults or 0.5 ml/kg/hr in children. Thus, in normal individuals, such urine output was achievable by urinary concentration, but, below this value, a decreasing urine output was linearly related to decreasing GFR as maximum urinary concentration had been achieved [14]. By the 1950s, these results had given rise to the concept that sustained oliguria implied a significant decrease in renal excretory function [15].

The concepts described above have been transferred almost unaltered into the RIFLE and AKIN contemporary consensus definitions of AKI [2,3]. In these systems, urine output of less than 0.5 ml/kg/hr for 6 or 12 hours is used to identify mild or intermediate kidney injury (RIFLE R or I; AKIN 1 or 2) and a urinary output less than 0.3 ml/kg/hr for more than 24 hours or anuria for more than 12 hours is taken to identify more severe AKI (RIFLE F; AKIN 3). The accuracy and usefulness of this urinary classification in real world clinical contexts, however, remains poorly understood. More relevant to this study, many other factors may modify the relation between urine output and GFR in critical illness. For example, in both acute and chronic renal disease, urinary concentrating capacity is often impaired [12] and urinary concentrating capacity is directly related to the kidney's ability to reduce urea clearance in relation to GFR [16]. Thus, although GFR may be significantly lower, the ability to generate a low volume of urine may be impaired. In these circumstances, a decline in GFR will ultimately result in a decrease in urine output, which may only meet conventional definitions of oliguria at very low levels of GFR making sustained oliguria a late and not early indicator of AKI. In addition, AKI-Cr can occur in the absence of oliguria [5,17]. Moreover, this relation may be frequently distorted by the administration of large amounts of intravenous fluids, diuretics, vasopressor drugs, or any combination of these. Conversely, oliguria can reflect salt and water retention as a normal renal response to a mild or moderate degree of hypovolemia or hypotension. Pain, trauma, and surgery can trigger similar neuro-endocrine responses resulting in oliguria in the absence of hemodynamic compromise [18-21] and in the presence of normal renal function [22].

Studies examining the application of sCr and urine output criteria in the AKIN and RIFLE definitions of AKI have shown that addition of the urine output criteria (oliguria ≥6 hours) to sCr criteria can improve the ability to predict mortality [23,24], but that urine output criteria alone show a lower predictive ability than sCr [25]. Macedo et al. [24] recently reported a prospectively single-center study of oliguria in 75 medical ICU patients, applying the AKIN criteria. In their study 24 (32%) patients had oliguria of six hours or more (< 0.5 ml/kg/hr) without developing AKIN-1 by sCr criteria while four patients (5%) developed sCr criteria without oliguria, and 17 patients (23%) had AKIN-1 AKI by both criteria. These results are broadly in agreement with the frequency of oliguria observed in our data and the notion that, while the majority of cases of AKI-Cr are associated with oliguria, significant periods (≥6 hours) of oliguria occur frequently without subsequent elevation of sCr even when using a much lower threshold for the diagnosis of AKI (AKIN-1). However, the above study did not assess the utility of oliguria as an earlier predictor of AKI-Cr and did not examine the ability of shorter periods of oliguria to predict AKI-Cr.

### Significance of study findings

Our study provides the only prospective multicenter assessment of oliguria in ICU to date. It shows that oliguria is, at best, only a fair predictor of subsequent AKI-Cr. Notably all short durations of oliguria (< 12 hours) were associated with positive likelihood ratios for AKI-Cr of only two to four. This finding suggests that the presence of these durations of oliguria during a given day does not usefully increase the post-test probability of AKI-Cr the next day, because, in general, a likelihood ratio of more than 10 is considered necessary for a clinically useful test [26,27].

Oliguria of 12 hours or more was associated with a high enough likelihood ratio to have clinical utility, in part validating its use in the RIFLE-Injury urine output definition. However, use of this cut-off would result in missing a large majority of cases of AKI-Cr. These observations are important because identifying patients at risk of developing AKI-Cr is the first and necessary step for clinicians to decide which patients should receive specific treatment and which patients should be observed. The crucial importance of these distinctions is supported by evidence that fluid resuscitation might reduce renal dysfunction [28], but that iatrogenic fluid overload can worsen outcomes [29-36] and the prognosis of AKI [37,38]. In addition, the early identification of patients at risk is vital because intervention only after biochemical evidence of AKI has developed (increased sCr) is likely to be too late. These clinical challenges are further complicated by the likelihood that the transition to renal dysfunction will not be abrupt. This gradual transition from appropriate physiological salt and water retention to injury is reflected in the limited diagnostic utility of urinary biochemistry in the early diagnosis of AKI the ICU [39].

Our data indicate that, if, for example, oliguria of two hours or more was used to trigger further fluid resuscitation, some patients might receive unnecessary fluid therapy, whereas others who might benefit from such intervention might not receive it. Thus, while clinically helpful, oliguria should be interpreted with caution in isolation, but may be more usefully interpreted in the clinical context as a screening test to direct additional methods for early detection of renal injury [40-42].

Another important observation from our study is that more patients had biochemically overt AKI on ICU admission than developed it in the ICU. This finding suggests the renal component of critical illness develops outside the ICU in most patients and that oliguria might be a more useful screening test in the pre-ICU environment. However, patients outside the ICU rarely have hourly documentation of urine output. Surgical patients may be more likely to have monitoring of urine output and develop AKI-Cr in the ICU. However, oliguria appeared, if anything, less predictive of AKI-Cr in this group - perhaps because transient postoperative oliguria is a common and relatively benign response to surgery.

### Study strengths and limitations

Strengths of this study include that it is the first study to investigate a common and important aspect of critical illness, its prospective design, and representation of a diverse population of critically ill patients from several countries and a variety of ICU settings. Thus, these results are likely to be widely applicable. On the other hand, a number of patients had their baseline sCr and/or body weight estimated, however, estimates are routinely used in clinical decisionmaking and such use seems appropriate when studying the real-world performance of oliguria and exclusion of all patient with estimated baseline creatinine did not materially affect the predictive ability of oliguria. Some clinicians might consider the choice of RIFLE I or greater as a threshold for diagnosis of AKI-Cr somewhat conservative, as smaller increments in serum creatinine may be associated with adverse outcomes. However, we wished to choose a severity of AKI-Cr that was clearly clinically significant and the potential target of interventional trials. The timing of observation with respect to the diagnosis of AKI-Cr could also be criticised. However, a rising sCr during a 24-hour period implies a fall in GFR. It is during this period that oliguria might be expected to occur most frequently and act as an early biomarker of patients with evolving AKI-Cr. Clinical management of patients was unaffected by any aspect of this study. Thus, episodes of oliguria may have triggered a variety of changes in treatment that might have averted the development of AKI-Cr. However, it would have been unethical to stop interventions in order to study the "natural history" of oliguria.

Univariate analysis suggested that patient characteristics such as hemodynamic status and physician response to oliguria were associated with the development of AKI-Cr; however, these factors are highly interdependent and the true significance of individual variables is difficult to assess. We were not able to better define the nature of these associations in multi-variate analysis as the small number of patients developing AKI-Cr and the high degree of co-linearity between hemodynamic variables and interventions preclude development of a statistically meaningful model from our dataset. Oliguria not associated with subsequent AKI based on sCr changes is likely still to be clinically significant and has been associated with adverse outcomes [23,24]. This may be related to better diagnostic sensitivity for the diagnosis of AKI or oliguria merely acting as a marker of global hemodynamic instability and illness severity. This study does not examine the wider implications of oliguria on outcomes or treatment and is limited to its association with AKI-Cr.

The results of this study should be taken not to dismiss, but to strengthen, the existing role of urine output in hemodynamic assessment and management in the ICU. However, they do indicate that, with standard treatment, most cases of oliguria are not associated with subsequent AKI-Cr in the ICU, and consequently, that trials of novel nephro-protective therapies, which aim to intervene early in the process of kidney injury should not use oliguria alone as a trigger for recruitment.

### Future studies

Use of oliguria alone may be insufficiently precise to be used as a trigger for intervention in trials of novel protective interventions. However, it may be more significant in the context of concomitant hemodynamic instability. Furthermore, the use of novel blood or urine biomarkers of AKI may increase diagnostic yield. Thus, future studies should focus on determining the combined predictive value of oliguria, clinical variables, standard urinary biochemistry, and novel biomarkers in the early diagnosis of AKI [40,43]. If this combination was shown to have good predictive value, it could be used as recruitment criterion in future early interventional trials of renal protection in the ICU.

## Conclusions

Oliguria is significantly associated with the occurrence of new AKI-Cr; however, most episodes of oliguria are not followed by biochemical evidence of renal injury. Oliguria alone is at best only a fair predictor of AKI-Cr. However, in the presence of hemodynamic compromise or increasing vasopressor dose, it may represent a clinically useful screening test to trigger other early biomarkers of renal injury with the goal of achieving a more accurate and timely identification of patients at risk of AKI.

## Key messages

• More patients develop AKI outside the ICU and present with it rather than developing AKI while in ICU.

• Using oliguria in isolation as a trigger for intervention in ICU might lead to some patients receiving unnecessary intervention and other patients not receiving potentially helpful intervention.

• Oliguria is relatively frequent in ICU patients and most episodes are not followed by AKI.

• Oliguria has only a fair predictive ability for subsequent AKI and lacks clinical utility as a test at the observed frequencies of AKI in the ICU.

• Oliguria accompanied by hemodynamic compromise or increasing vasopressor dose may represent a clinically useful trigger for other early biomarkers of renal injury with the goal of achieving a more accurate and timely identification of patients at risk of AKI.

## Abbreviations

AKI: acute kidney Injury; AKI-Cr: acute kidney injury defined by changes in serum creatinine; AKIN: Acute Kidney Injury Network; AUC: area under the curve; CI: confidence interval; GFR: glomerular filtration rate; IQR: interquartile range; RIFLE: Risk Injury Failure Loss End stage; RIFLE-I: RIFLE-Injury: RIFLE I[Cr]: RIFLE-Injury by serum creatinine criteria; ROC: receiver-operator characteristic; SAPS II: Simplified Acute Physiology Score II; sCr: serum creatinine; SIRS: systemic inflammatory response syndrome.

## Competing interests

The authors declare that they have no competing interests.

## Authors' contributions

JRP participated in study design, collected data, prepared data, performed statistical analysis and wrote the paper. YL and EL participated in study design and collected data. SM collected data and was the center organiser (Edmonton). ME collected data and was the center organiser (Okayama). MH and AH-F collected data and were center organisers (Berlin). JK was the center organiser (Pittsburgh). CR and DC collected data and were center organisers (Vicenza). KT collected data and SU was center organiser (Jikei). RB conceived the study, coordinated study centers, participated in study design, and edited the final manuscript. All authors read and approved the final manuscript.
